# A systematic review of measures of the personal recovery orientation of mental health services and staff

**DOI:** 10.1186/s13033-023-00600-y

**Published:** 2023-10-17

**Authors:** Mary Leamy, Una Foye, Anne Hirrich, Dagfin Bjørgen, Josh Silver, Alan Simpson, Madeline Ellis, Karl Johan-Johanson

**Affiliations:** 1 Florence Nightingale Faculty of Nursing, Midwifery and Palliative Care, King’s College London, London, UK; 2 KBT Competence Center for lived experience and service development, Trondheim, Norway; 3 London, UK

**Keywords:** Systematic review, Recovery, Measure, Reliability, Validity, Mental health services.

## Abstract

**Purpose:**

This review aimed to update and extend the Williams and colleagues 2012 systematic review of measures of recovery-orientation of mental health services by examining whether any of the specific knowledge gaps identified in this original review had subsequently been addressed.

**Methods:**

A systematic review using CINAHL, ASSIA, Embase, PsycINFO, Medline and other sources, searched from 2012 until 2021. The conceptualisation of recovery and recovery-orientation of services was explored. Psychometric properties of measures were evaluated using quality criteria and according to ease of use.

**Results:**

Fourteen measures assessing aspects of the recovery orientation of services and staff were identified, of which ten met the eligibility. Psychometric properties were evaluated, and conceptualisations of recovery and recovery-orientation of services investigated.

**Conclusion:**

After over a decade of research in the field of recovery outcome measurement, there remains a lack of a single gold-standard measure of recovery-orientation of mental health services. There is a need for researchers to develop a new gold standard measure of recovery-orientation of services that is psychometrically valid and reliable, demonstrates sensitivity to change and is easy to use. It needs to show a good fit to an underpinning conceptual model/ framework of both personal recovery *and* recovery-oriented services and/or systems, with different versions for stakeholders at each level of an organisation or system.

**Supplementary Information:**

The online version contains supplementary material available at 10.1186/s13033-023-00600-y.

## Introduction

### Mental health policy and practice

Transforming mental health services to focus on supporting the personal recovery of mental health service users has now been part of mental health policy across the Western world and in Australia and New Zealand for some time [1, 2]. The personal recovery concept and personal recovery-oriented practice inventions are increasingly being incorporated into mental health policy and practice in Asian countries [3, 4]. There are predominately two arguments for transforming mental health services towards a personal recovery-orientation. Firstly, longitudinal research led to a growing recognition that full clinical recovery is possible from an acute episode of mental illness [5]. Secondly, it is grounded within research into the narratives of the lived experiences of mental health consumers/survivor/service user movement in mental health, describing how people with lived experience of mental health problems understand recovery and what has helped them with their recovery, moving beyond a patient role/identity to a fully contributing citizen [6, 7].

### Personal & clinical recovery

Personal recovery has been characterized as a deeply personal process, defined by persons with lived experience as ‘a way of living a satisfying, hopeful, and contributing life’ even with any limitations caused by illness [8]. It involves the five recovery processes of Connectedness, Hope and optimism about the future, Identity, Meaning and purpose in life and Empowerment (CHIME) [9]. Personal recovery is distinct from clinical recovery, which focusses upon the cure-oriented concept of “recovery as remission of illness”, which has been the traditional view of recovery, grounded within a medical model [10].

In the last decade, Personal Recovery has been related to concepts of citizenship, defined as the extent of how connected people are to the “rights, responsibilities, roles, and resources that society offers to people through public and social institutions, and relationships involving close ties, supportive social networks, and associational life in one’s community” [11, 12].This overlaps with the mental health consumer/ mental health survivor movement’s view of recovery. Newer developments in this field emphasise the importance of social and relational processes in recovery, for instance studies examining the mediating role of mattering to others in recovery, where adults living with a serious mental health condition feel isolated, stigmatised and that they do not matter to others [13]. There have been critical conceptual critiques of personal recovery theory for being reductionist and its lack of focus on the socio-structural inequalities on the recovery processes, such housing, ethnicity, gender, socio-economic status [14].

In a recent scoping review of conceptualisations of personal recovery, Van Weeghel and colleagues [15] whilst stating that the CHIME framework was widely endorsed, did highlight some critiques and limitations. They suggested that CHIME framework should be adapted according to culture and unique population characteristics, that difficulties and trauma should be added and that a person’s choice, risk-taking and coping with challenges should be emphasised. Empirical studies have compared personal and clinical recovery outcomes and demonstrated that they are conceptually different and not necessarily associated [16, 10, 17]. Therefore, traditional, symptom focused treatment is unlikely to be sufficient and to achieve recovery-oriented mental health services and staff need to offer interventions and recovery support which target both clinical and personal recovery outcomes [17].

### Recovery-oriented mental health services

The purpose of transforming mental health services towards a recovery-oriented approach is to support people to create and sustain a personally meaningful and satisfying life and personal identity, with or without experiencing ongoing symptoms of mental illness [18]. The term ‘recovery-oriented practice’ describes this approach to mental health care, which incorporates the principles of self-determination and personalised care, and emphasises hope, social inclusion, community participation, personal goal setting and self-management. Boutillier and colleagues reviewed international guidance on recovery-oriented practice to identify the key characteristics of recovery-oriented practice and to develop an overarching conceptual framework to aid the translation of recovery guidance into practice [19]. The emerging conceptual framework consists of 16 dominant themes, grouped into four practice domains: (i) Promoting citizenship through having a meaningful occupation, promoting service user rights and social inclusion and seeing beyond “service user”; (ii) Organizational commitment, through having a recovery vision, workplace support structures, quality improvement, care pathways, and workforce planning that are geared towards supporting recovery; (iii) Supporting personally defined recovery, through emphasizing individuality, informed choice, strengths focus and holistic approach and (iv) Working relationship, through developing partnerships and inspiring hope.

Typically, literature on recovery-oriented practice promotes a coaching or partnership relationship between [20]people accessing mental health services and mental health professionals, whereby people with lived experience are considered experts on their lives and experiences while mental health professionals are considered experts on available treatment services. In the recent Australian mental health framework, recovery-oriented practice is understood as encapsulating mental healthcare that (i) encourages self-determination and self-management of mental health and wellbeing; (ii) involves tailored, personalised and strengths-based care that is responsive to people’s unique strengths, circumstances, needs and preferences; (iii) supports people to define their goals, wishes and aspirations; (iv) involves a holistic approach that addresses a range of factors that impact on people’s wellbeing, such as housing, education and employment, and family and social relationships; and (v) supports people’s social inclusion, community participation and citizenship. Citizenship in this context is understood to refer to people’s full inclusion and participation in all aspects of public, social and cultural life [21]. Davidson and colleagues et al. developed an inventory of transformation characteristics for a recovery- oriented system of care, describing what systems look like pre and post transformation, which promotes recovery and citizenship [22]. The inventory has four over-arching characteristics: i) How people receiving health services are viewed and treated by staff, ii) How people receiving health services are included in the design, delivery, and evaluation of care, iii) How care is planned, delivered, and improved on a continuous basis, and iv) How transformation is led and managed.

The purpose of the present study was to update and extend Williams and colleagues systematic review about measures of the recovery orientation of mental health services, which was conducted over a decade ago, in 2012 [23]. The aims of the Williams and colleagues systematic review were threefold: (i) To identify measures that assess the recovery orientation of services, (ii) To discuss how these measures have conceptualised recovery, and (iii) To characterise their psychometric properties. This review used seven sources and conceptualised recovery using the CHIME personal recovery framework and evaluated the psychometric properties of measures using quality criteria. The review identified thirteen recovery measures, of which six met the eligibility criteria and concluded that none of the measures had undergone sufficient psychometric and sensitivity testing. It also found that the six measures varied considerably in the ways personal recovery had been conceptualised and the organisational level of services, making it hard for services and researchers to decide which was the best measure to select.

### Aims and objectives

There were two overarching and related aims of the present review. The first aim was to update the Williams and colleagues review by examining whether any of the specific knowledge gaps identified in this original review had subsequently been addressed, and specifically if and how this new knowledge might be helpful to potential users of measures of recovery orientation of services and staff (such as persons with mental health problems, staff, leaders and researchers) in informing their choice of measures. The second aim was to extend the Williams and colleagues review [20].

To address the first aim, the review was updated by broadening the eligibility criteria to include: (i) measures of recovery orientation of mental health services and staff published after 2012, (ii) all adults, rather than just working age adults, (iii) service user rated measures that assess the contribution of individual staff to supporting their recovery, and (iv) staff rated measures of recovery knowledge, attitudes, recovery-promoting relationships and competencies. The Williams and colleagues review excluded measures of the knowledge, attitudes, competences of individual staff members, or recovery-promoting relationships that promote or hinder recovery [20]. It is assumed that both providers and programmes that are not promoting recovery in their work with service users become barriers which hinder the recovery process [24, 25]. In the field of mental health, Bledsoe and colleagues identified provider characteristics that were recovery-facilitating such as being hopeful, positive, and holding a belief that recovery is possible, and recovery-hindering such as having low expectations and negative attitudes [26]. As with the previous review, measures relating to recovery in substance use or relating to children and adolescents are excluded.

To address the second aim, the review examined the ways in which both ‘personal recovery’, *and* ‘recovery-oriented practices’ are conceptualised and operationalised in research measurement instruments. The original review did not include an analysis of how the measures conceptualised recovery-orientation of services.

The overarching aims have been operationalised as five objectives.

### Objectives

(i) To identify the standardised, service-user rated measures that assess the contribution of mental health services and individual staff in supporting personal recovery;
(ii) To identify the standardised, staff rated measures of recovery-orientation of mental health services, recovery knowledge, attitudes, recovery-promoting relationships and competencies;
(iii) To assess how these measures conceptualise recovery;
(iv) To assess how do these measures conceptualise recovery-orientation of mental health services/ systems;
(v) To assess the psychometric properties of these measures and how easy are they to use.

## Methods

The study was reported in accordance with the Preferred Reporting Items for Systematic Reviews and Meta-Analyses (PRISMA) guidelines [27].

### Search strategy

Searches were conducted using the following databases: MEDLINE, PsycINFO, EMBASE, CINAHL and ASSIA. Each database was searched from 2012 until 2021.The search terms used for the electronic databases were divided into four domains, personal recovery, mental illness, measure or instrument and psychometric properties. These terms were identified from the title, abstract, keywords, or medical subject headings (MeSH). The terms were modified for each database as needed. The full search terms are provided in a supplementary file. Second, web searches were undertaken using Google Scholar (‘recovery’ AND ‘mental health’ AND ‘measure’) and measures mentioned in literature reviews published 2012 or after were considered, and reference lists of all included articles were hand searched.

### Eligibility criteria

Measures were included if they met the thirteen inclusion criteria: 1) Service user (mental health problems); 2) Staff working in mental health services; 3) All adults; 4) Measures that assess the contribution of mental health services in supporting personal recovery of service users with mental health problems; 5) Measures that assess the contribution of mental health staff to support personal recovery of service user with mental health problems; 6) Outcomes related to support for Personal Recovery for mental health problems; 7) Has a version of measure rated by service users and/or staff; 8) Measure produced quantitative data; 9) Measure and at least one associated psychometric paper were published and obtainable; 10) Written in English; 11) Peer reviewed; 12) Published on or after 2012 and 13) Measure is freely/publicly available.

Measures were excluded if they met the two exclusion criteria: a) Measures that assess the experience of personal recovery, rather than the contribution of services or staff to recovery; or b) They were any of the following: Commentaries, discussions, editorials, policy papers, grey literature, PhDs and systematic reviews of recovery measures.

### Data extraction

We imported all articles that were identified using our search terms into Covidence software, then these were all screened by at least two reviewers who rated articles on basis of title/abstract as being relevant as ‘yes’, ‘no’ or ‘maybe’ (ML, UF, ME). Articles rated as ‘maybe’ were considered by a third reviewer and any disagreements were discussed with a lead author. A concordance level of 90% was considered acceptable and this was achieved. Where the abstract appeared relevant, the full text of the paper and associated measure was obtained. Following review of the paper, a decision was made on including the measure.

A data extraction spreadsheet was created to consistently document data from each of the articles, conducted by lead author and co-authors. The data extraction spreadsheet had columns to extract data on authors/date/country, aims and objective/s, intended population, study design, stakeholder/service user involvement in development of measure. It also had a section for information on the findings related to coverage of conceptualisation of recovery processes (CHIME), transformation characteristics of recovery-oriented systems of care, and psychometric properties.

No available quality appraisal tool for assessing psychometric properties of measures was identified. Measures were compared in on key psychometric properties of content, construct, convergent validity and internal consistency, test-retest reliability and sensitivity to change, following the types of validity and reliability selected in the previous Williams et al review, along with a proxy indicator of ease of completion, namely the time to complete the measure.

## Results

### Objectives 1 and 2 (identifying measures that met the eligibility criteria)

**Fig. 1:**
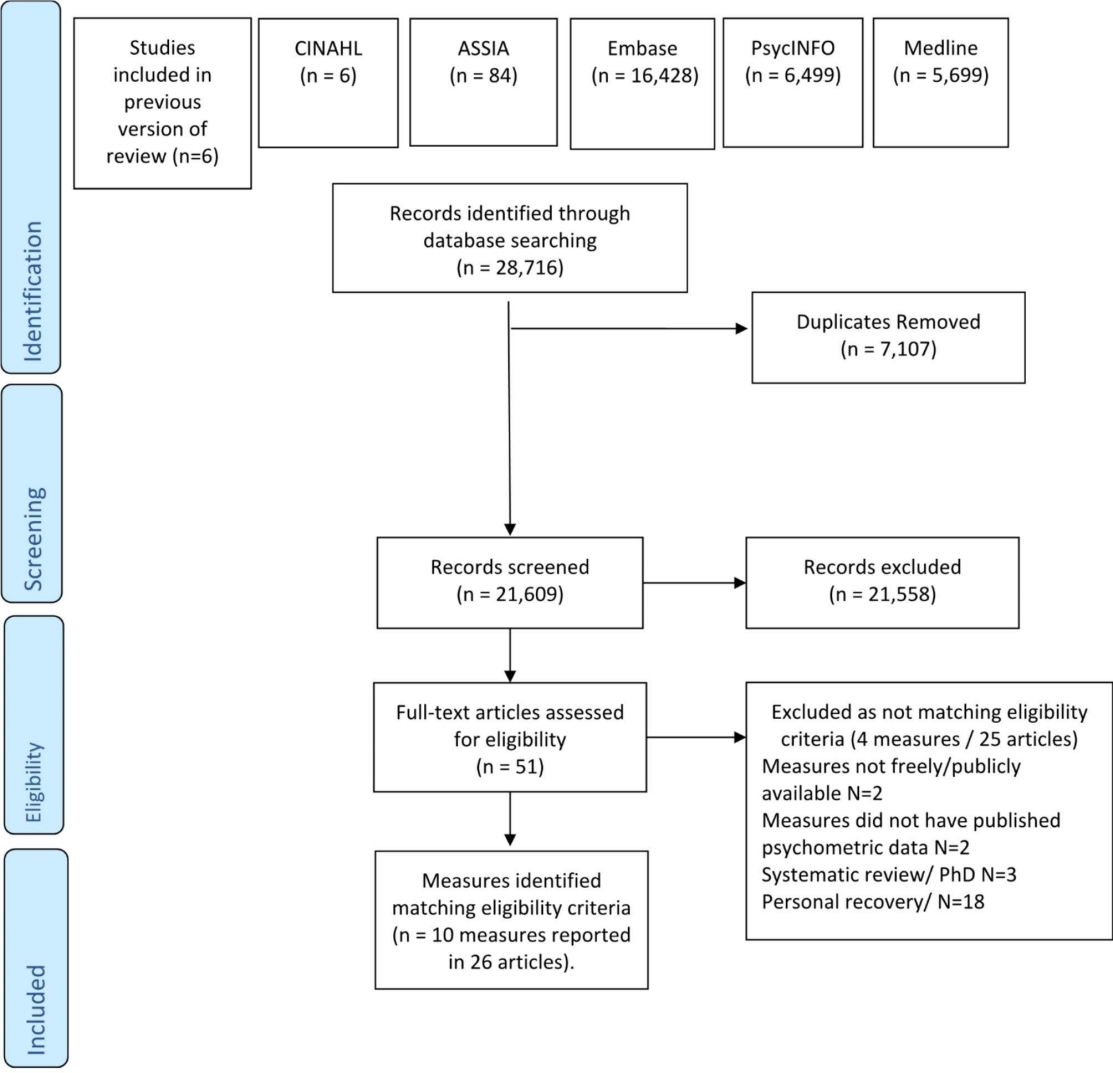
PRISMA Flow Diagram for review of measures

This review identified fourteen measures assessing aspects of the recovery orientation of services, ten of which met the eligibility criteria, reported in 26 articles, as shown in Prisma flow diagram in Fig. 1. Four measures did not meet the inclusion criteria and were excluded, for the following reasons: Measures not publicly or freely available (Therapeutic Engagement Questionnaire, [28], Illness Management Recovery – Treatment Integrity scale [29]. The TEQ is currently under further development, but there are plans to make the measure publically available in the future^1^. No published psychometric data (Strengths Model Fidelity Scale; Illness Management and Recovery Program Fidelity Scale, [30].

This current review identified two new standardised, service-user rated measures that assess the contribution of individual staff or services in supporting personal recovery, developed since the original article was published. These were the INSPIRE measure, which is a measure of staff support for recovery, rated by service users [31]; and the RECOLLECT measure, which is a fidelity measure to evaluate Recovery Colleges, with different versions rated by service user students, peer trainers, Recovery College managers [32].

Of the six measures that met the original inclusion /exclusion criteria in the Williams and colleagues review, three measures: Recovery Enhancing Environment (REE), Recovery Self-Assessment (RSA) and an adaptation of the RSA called Recovery-Oriented Services Assessment (ROSA) are also included in this review because they have now been translated, validated and psychometrically tested for use with different populations and settings.

Extending the inclusion criteria from the original Williams et al. paper to ‘standardised, staff rated measures of recovery-orientation of mental health services, recovery knowledge, attitudes, recovery-promoting relationships and competencies’, has led to the inclusion of five additional measures: Recovery Knowledge Inventory (RKI), a staff rated measure of recovery knowledge and understanding, originally developed in Bedregal and colleagues [33]; Recovery Attitudes Questionnaire (RAQ), a staff rated measure which assesses attitudes about the belief that people can recover from mental illness, originally developed by Borkin and colleagues [34]; Attitudes towards Recovery Questionnaire (ARQ), a measure of attitudes towards recovery, developed and tested with service users, carers, service providers; Provider Expectations for Recovery Scale (PERS), a staff rated measure of their expectations of the numbers of service users on their caseloads that they expected to have recovery-related outcomes; and Recovery Promoting Relationships Scale (RPRS), a measure of mental health providers’ recovery-promoting competence.

The PRISMA flow diagram, set out in the PRISMA statement in Fig. 1 identifies search process [35], and a summary of key characteristics of included measures is presented in Table 1.

**Table 1:** Brief summary of key characteristics of included measures

#	Name of measure	Versions available	Items	Constructs assessed	Country of origin	Primary reference
1	Attitudes towards Recovery Questionnaire (ARQ)	Service users, carers, service providers	18 items	Attitudes towards recovery	China	Mak et al, (2018).
2	INSPIRE	Service user	i)Full INSPIRE (27 items) ii) Brief INSPIRE (5 items) iii) INSPIRE-O (5 items)	INSPIRE and Brief INSPIRE assess recovery support of an individual worker INSPIRE-O assesses recovery.	UK	Williams et al, (2015)
3	Provider Expectations for Recovery Scale (PERS)	Service providers	10 items	Expectations of the numbers of service users on caseload that are expected to have recovery-related outcomes	USA	Salyers et al, (2013)
4	Recovery Attitudes Questionnaire (RAQ)	Service providers	7 items	Attitudes about the belief that people can recover from mental illness	USA	Borkin et al, (2000).
5	Recovery Knowledge Inventory (RKI)	Service providers	20 items	Knowledge of and attitudes toward recovery-oriented practices	USA	Bedregal et al, (2006)
6	Recovery Promoting Relationships Scale (RPRS)	Service providers	38 items	Mental health providers’ recovery-promoting competence	USA	Russinova et al, ( 2013)
7	Recovery -Oriented Services Assessment** (ROSA)	Service provider, People in services	ROSA = 15 items	Recovery-oriented services	USA	Lodge et al, (2018)
8	Recovery Self-Assessment* (RSA)	Person-in-recovery, significant other, service provider, service director	RSA = 36 items; Brief RSA (RSA-B) = 12 items;	Extent of recovery-supporting practices	USA	O’Connell et al, (2005)
9	Recovery Enhancing Environment Measure* (REE)	Service user	REE = Up to 166 items for some particular groups	Service contribution to recovery and organisational climate as well as other aspects of recovery	USA	Ridgeway et al, (2004)
10	RECOLLECT	Service user students, peer trainers, Recovery College managers	RECOLLECT = 12 components, 7 non-modifiable and 5 modifiable components.	Fidelity of recovery colleges.	UK	Toney et al, (2019).

*Original measure included in Williams et al, 2012.

** Revision of service provider version of RSA.

### Objective 3 (assessment of how measures conceptualise recovery)

Items were allocated to CHIME personal recovery processes by two raters, using the full personal recovery coding framework (ref CHIME). Raters determined whether or not the item contained a CHIME recovery process or not, and recorded which process that was, and any disagreements were discussed. Rater disagreement arose due to the following reasons: an item asking more than one question; a lack of clarity in the wording of the item; an item that covered more than one or none of the CHIME personal recovery processes. Table 2 shows the coverage of each CHIME recovery process and other constructs which were operationalised in the measures.

**Table 2a:** Coverage of CHIME conceptual framework of personal recovery processes for measures of recovery-orientation of individual staff/workers

#	Measure	Total number of items	Connectedness	Hope and optimism	Identity	Meaning and purpose	Empowerment	Other constructs/ Items not mapping
1	Attitudes towards Recovery Questionnaire (ARQ)	18 items	7 (39%)	2 (29%)	4 (22%)	1 (6%)	5 (28%)	Items relating to ‘Involvement of family in care plans’.
2	INSPIRE	28 items	4 (20%)	4 (20%)	4 (20%)	4 (20%)	4 (20%)	None
3	Provider Expectations for Recovery Scale (PERS)	10 items	4 (40%)	0	0	3 (30%)	0	Items relating to functional recovery
4	Recovery Attitudes Questionnaire (RAQ)	7 items	0	4 (57%)	0	0	1 (14%)	Items relating to recovery characteristics rather than processes
5	Recovery Knowledge Inventory (RKI)	20 items	1 (5%)	2 (10%)	1 (5%)	1 (5%)	5 (25%)	Items relating Recovery-readiness, managing symptoms, Individual process, Incorporating illness, Non-linear process.
6	Recovery Promoting Relationships Scale (RPRS)	24 items	0	10 (26%)	7 (18%)	10 (26%)	10 (26%)	Items relating to ‘Quality of the core relationship between provider and service user’.

**Table 2b:** Coverage of CHIME conceptual framework of personal recovery processes for measures of the recovery-orientation of mental health services/systems

#	Measure	Total number of items	Connectedness	Hope and optimism	Identity	Meaning and purpose	Empowerment	Other constructs/ Items not mapping
7	Recovery -Oriented Services Assessment (ROSA)	15 items	3 (20%)	1 (7%)	2 (13%)	7 (47%)	2 (13%)	None
8	Recovery Self-Assessment* (RSA)	30 items	5 (20%)	2 (8%)	3 (12%)	5 (20%)	10 (40%)	11 items
9	Recovery Enhancing Environment Measure* (REE)	43 items	8 (22%)	3 (8%)	4 (11%)	10 (27%)	12 (32%)	Items that related to sub-scales.
10	Recollect	12 items	2 (16%)	1 (8%)	3 (25%)	2 (16%)	5 (42%)	

Each measure is based on different conceptualisations of recovery, with the items consequently including broader definitions of recovery, namely clinical recovery, and functional recovery. Some items which did not map onto the CHIME framework of personal recovery processes related to clinical recovery (e.g., RKI’s items on managing symptoms; PERS’s items on staff expectations regarding medication use, drug and alcohol use, or functional recovery (e.g., PERS’s items on social and occupational resumption of functional recovery such as housing and competitive employment) [26]. Other items related to the characteristics of recovery (e.g., RKI’s items on recovery being a non-linear process, individual process, recovery is difficult and differs among people).

One recovery measure of the recovery-orientation of staff was culturally adapted for non-westernised culture. The ARQ measure was developed in Hong Kong, in recognition that the conceptualisation of personal recovery is likely to vary based on the cultural context, and that ‘a measure needs to take into account indigenous values that are unique to the cultural milieu as well as universal values of recovery’ [36], (page 49). The ARQ measure development was based on core recovery values and contribution of family involvement in process of recovery, and grounded in a literature review of empirical studies, focus groups of key stakeholders in mental health system, and from reviewing existing measures of personal recovery. The measure included items which are emphasised in the Chinese culture, for example, a strong emphasis on familism and close kin relations [37]. The ARQ measure consists of five domains: i) resilience as a person in recovery, ii) self-appreciation and development, iii) self-direction, iv) family involvement, v) social ties and integration. Since the Williams and colleagues review, some measures have since been psychometrically validated in different cultures and populations (see below - psychometric properties of measures section).

### Objective 4. (assessment of how measures conceptualise recovery-orientation of mental health services and systems).

Items were allocated to the inventory of transformative characteristics of recovery-oriented systems of care framework by two raters (insert REF). Rater agreement was highest for the items where there was either no items or only one item that related to a characteristic of recovery-oriented systems of care. Rater disagreement arose because some items only implied recovery-oriented characteristic rather than clearly stated this, and some items were very broad and subject to interpretation. In reaching an agreement, and to be consistent, all items that at least one of the raters considered contained a characteristic was counted.

The conceptualisation of the recovery-orientation of mental health services was not fully developed for any measure, the RSA and RECOLLECT measures had the most well-developed conceptual foundations, being based on empirical research.

Table 3 shows how the items from the four measures of recovery-oriented mental health service items (RSA, ROSA, REE and RECOLLECT) could be categorised according to the inventory of transformative characteristics of recovery-oriented systems of care framework. The table shows how each of the measures operationalised the features of a recovery-oriented service and system of care. The RSA measure most closely matched the characteristics of the inventory, with three or more items relating to: ‘People receiving health services are viewed and treated as unique individuals’, ‘People having a voice in the system’, ‘Input being sought from service users and families’, ‘Leadership emphasizes shared decision-making and collaborative care’.

**Table 3:** Coverage of inventory of transformation characteristics of recovery-oriented systems of care for measures of recovery-orientation of mental health services

Transformation characteristics of recovery-oriented systems of care	Recollect	REE (Organisational climate)	ROSA (Revision of RSA provider version)	RSA-R (provider version)
How people receiving health services are viewed and treated by staff				
1. People receiving health services are viewed and treated as unique individuals	5 items	1 item	7 items	6 items
2. Human rights are respected	1 item	1 item	1 item	3 items
3. Staff know about and expect recovery	all items	None	3 items	All items
How people receiving health services are included in the design, delivery, and evaluation of care				
4. People in recovery have a voice in the system	1 item	1 item	None	8 items
5. Recovery advocacy community is a valuable ally	1 item	None	1 item)	1 item
6. Input is sought from service users and families	1 item	3 items	3 items	9 items)
7. Peer supports are integrated	3 items	None	1 item	2 items
How care is planned, delivered, and improved on a continuous basis				
8. Focus of care is on building a healthy and self-determined life in the community	2 items	None	1 item	7 items
9. Care is community-based and focused	3 items	None	1 item	4 items
10. There are trauma-informed crisis alternatives	None	None	1 item	None
11. Community life is encouraged	2 items	None	None	5 items
12. Hope is instilled	all items	1 item	3 items	2 items
13. Care plans are based on each person’s life goals	None, but related 4 items	None	6 items	3 items
14. Coercion is avoided	None	None	1 item	1 item
15. Access to trusting relationships is emphasized	1 item	None	None	1 item
16. Trauma is addressed	None	None	1 item	None
17. Families are involved	None	None	1 item	1 item
18. Outcomes are assessed	None	None	None	1 item
19. QI and PM results are used to improve quality of care	None	2 items	None	None
20. Physical health is attended to	1 item	None	None	None
21. Attention is paid to enhancing social support	1 item	1 item	None	2 items
22. Staff pay attention to basic needs and social roles	2 items	None	None	3 items
23. Staff address social & economic health determinants	None	None	None	None
24. Staff collaborate with clients in addressing social & economic barriers	None	None	None	None
25. Disparities are addressed	1 item	None	2 items	2 items
26. Staff ask for feedback	None	2 items	None	1 item
27. System educates youth, adults, and family members on self-care	1 item	None	None	None
How transformation is led and managed				
28. Leadership are engaged and action-oriented	None	None	None	None
29. Leadership are strength-based and encourage risk taking	1item	None	1 item	1 item
30. Leadership emphasizes shared decision-making and collaborative care	1 item	1 item	4 items	3 items
31. Workforce has been trained in recovery-oriented care	all items	None	None	None
32. Workforce is culturally responsive	2 items	None	2 items	5 items

### Objective 5. (assessment of the psychometric properties of these measures and how easy are they to use).

The current review identified nine articles which either provided additional data on the psychometric properties or shortened or adapted version of two of the original six measures. The RSA measure has been revised in several ways. The provider version of RSA has been revised and renamed as the ROSA [38], and adapted for a specific group of providers – the RSA Registered Nurse version (RSA-RN) [39]. It has also been tested in two Asian countries. There is now a RSA Chinese translation which was validated in Hong Kong (RSA – HK) [40], a Chinese RSA Service User version (CRSA-SU) [41], and a Persons in Recovery Version Malay RSA (PIRV- RSA) [42]. It has also been psychometrically tested in Canada and named as the Revised RSA (RSA-R) [43], and in Sweden, (RSA-S) [44], and there is now a validated brief version of the RSA Validation (RSA-B) [45]. The REE has been adapted and had additional Psychometric testing, for use in Spain [46].

### Content validity

The majority of measures had involved service users in the development of the measure, as consumer researchers (INSPIRE, REE) and/or advisory experts (RAQ, ARQ, RPRS, RECOLLECT, RSA, ROSA). Two measures (RKI, PERS) did not have service user involvement. The RKI was developed using a definition which combined clinical and personal recovery, based on the expertise of the authors, identifying the following issues most integral to the provision of clinical and rehabilitative services oriented to promoting recovery: consumer directedness, the individual nature of recovery, cultural competence, self-determination, strengths-based care, choice and risk-taking, illness and symptom management, incorporation of illness into sense of self, involvement in meaningful activities, overcoming stigma, redefining self, hope, and the non-linear nature of the recovery process. The PERS measure was originally developed as a 7-item Optimism scale by Grusky and colleagues [47], then expanded into a 16-item Consumer Optimism scale by Sayers and colleagues [48]. It was renamed the Provider Expectations for Recovery Scale to better capture the construct.

### Cross-cultural validation

Some measures have now been tested outside their country of origin. These include the RSA which has been tested in China - Hong Kong, with a RSA – HK version [40] and CRSA-SU version [41] and Malaysia - with a Persons in Recovery Version Malay RSA (PIRV- RSA) [42], in Canada (RSA-R) [43], and Sweden, (RSA-S) [44]. The Spanish adaptation and translation of REE was psychometrically tested in a representative sample of 312 people with severe mental health disorders [46]. Each section of the REE (importance of recovery elements, experience of recovery elements, organizational climate and recovery markers) showed unidimensionality of the scale, with suitable indexes in the factorial analyses and Cronbach alphas greater than 0.90 for each dimension.

### Reliability

All measures had been subject to at least one type of reliability test, with Cronbach alpha’s internal consistency being the most frequently used, which ranged from acceptable internal consistency (α = 0.7 for RKI, RECOLLECT, RAQ) to high internal consistency (α 0.9 = RSA, REE, RPRS, INSPIRE, ARQ).

The psychometric properties of the ten measures are shown in Table 4.

**Table 4:** Psychometric properties

#	Measure			Conceptual model			Content validity		Construct validity		Convergent validity		Internal consistency	Test-retest consistency		Sensitivity to change			Completion time	
1	Recovery Knowledge Inventory (RKI, 20 items, no sub-scales)			RKI designed to include Clinical recovery and Personal recovery.			Yes		Not correlated with other measures. Egeland and colleagues challenged previous four factor structure of RKI. Happell et al, 2015 argue conceptual underpinnings need reworking to improve reliability and validity of RKI (56).		Unknown		Reliability analysis (Cronbach’s alphas) estimates for the four components were .81, .70, .63, and .47, (33). Cronbach alpha 0.72 (63).	Unknown			Unknown			15 minutes
2	Recovery Attitudes Questionnaire (RAQ. 7 items, no sub-scales)			RAQ designed to measure whether respondents believed people with mental illness could recover			Yes. Developed with service users, mental health professionals		Unknown		Unknown		Reliability analysis (Cronbach’s alpha) for RAQ-7 was 0.704. Factor 1 (Recovery is possible and needs faith) − 0.655; Factor 2 (Recovery is difficult and differs among people) − .0644	Test-retest reliability coefficients 0.674 for RAQ – 7; 0.609 factor 1 and factor 2 0.619.			Unknown			5 minutes
3	Attitudes towards Recovery Questionnaire (ARQ, 18 items, no sub-scales)			ARQ based on reviewing literature and existing measures, focus group discussions with service users, carers, staff.			Yes. Items reviewed by expert panel (service users, clinicians)		Yes. All five factors of the ARQ were positively and moderately associated with the Recovery Markers Questionnaire in the people in recovery sample (rs ranged from 0.31 to 0.46, p < .001).		Unknown		Cronbach’s alphas = 0.87 in the people in recovery sample, 0.90 in the carer sample, and 0.95 in the service provider sample.	Unknown			Unknown			Unknown
4	Provider Expectations for Recovery Scale (PERS, 10 items, no sub-scales)				PERS assesses optimism about consumers’ recovery-related outcomes.		Yes		Construct related to optimism about role functioning. PERS renamed 16 item Consumer Optimism Scale.		Convergent validity: PERS and Burnout subscales; lower levels of emotional exhaustion (r = 0.27) depersonalization (r = 0.29), greater sense of personal accomplishment (r = 0.37).		High internal consistency (α = 0.91)	High test-retest consistency over 2 weeks (r = 0.92)		Unknown			5 minutes	
5	Recovery Promoting Relationships Scale (RPRS, 24 items, 3 sub-scales: Hopefulness, Empowerment and Acceptance)				RPRS based on reviewing literature and clinical experience. Designed to measure ways service user’s hopefulness and empowerment can be enhanced by clinicians		Yes. Relevance of items to recovery assessed by service users, peer-providers, and providers explored via survey.		Exploratory and confirmatory factor analysis. Four factors: Hope, Empowerment, acceptance and Core relationship – reduced number of items from 38 to 24 with an acceptable fit.		Correlation of the overall RPRS with the overall Working Alliance Inventory suggests good convergent validity of 0.79.		High, 0.88 to 0.98 internal consistency.	Acceptable test–retest reliability. Coefficients of stability ranged from .61 to .72 for the total score and .75 for the Core Relationship Index		Unknown			Unknown	
6	INSPIRE (27 items, 2 sub-scales: Support and Relationships)			INSPIRE based on a CHIME recovery processes and a review of best practice of recovery-oriented practice.			Yes, developed with Service users.		Exploratory factor analysis supported 5 factor solution for CHIME domains of support sub-scale. Parallel analysis supported one factor solution for the relationship sub-scale.		Adequate convergent validity between Relationship sub-scale and RPRS (r = 0.69) Low convergent validity between Support sub-scale and the SIMH*** (r = 0.47).		Relationship sub-scale, (α = 0.89) Good Support sub-scale, internal consistency was calculated for each CHIME domain. 0.82 (Identity), 0.83 (Hope), 0.84 (Connectedness), 0.85 (Meaning), adequate internal consistency. 0.95(Empowerment)	7 item relationship sub-scale test-retest reliability = 0.75			Adequate			7 minutes
7	Recovery -Oriented Services Assessment (ROSA, 15 items, no sub-scales)				ROSA based on revision of RSA (revised) provider version.		Developed with feedback from expert peer provider consultants to provide brief 15 item tool, with more recovery-oriented language than RSA.		Exploratory factor analysis supported a one-factor solution.		Unknown		Unknown	Unknown			Unknown			Unknown
8	Recovery Self-Assessment (RSA, 30 items, 5 subscales: Life goals, Involvement, Diversity of treatment options, Choice and Individually-tailored services)				RSA based on literature reviews, recovery principles of empowerment, stakeholder involvement.		Developed with feedback from service users.		Unknown		Unknown		0.93–0.94	Unknown		Unknown			10 minutes	
9	Recovery Enhancing Environment Measure (REE, 43 items, 4 sub-scales: importance of recovery/experience / organizational climate/ recovery markers				Based upon: consumers’ first person accounts of their recovery and the supports that assisted them in this process; an informal review of promising practices; and a review of literature on factors that promote resilience or “rebound from adversity” .		Unknown		Unknown		Unknown		0.94–0.97	Unknown		Unknown			40 minutes	
10		RECOLLECT checklist and fidelity measure (12 items, no sub-scales)	Based on literature review, expert consultation, semi-structured interviews with recovery college managers.			Developed with experts, peer trainers, recovery college managers, service users		Item hierarchy (ie. Construct validity) in terms of how easy to endorse (from highest to lowest). Co-production, Learning, Available to all, Strengths based, Distinctiveness of courses, Location.		Unknown		α = 0.72		Test-retest intraclass correlation coefficients = 0.6				Unknown		

## Discussion

### Identification of measures

Fourteen measures assessed the recovery orientation of mental health services and staff, and of these ten measures matched the eligibility criteria for inclusion. Two measures were new standardised, service-user rated measures that assess the contribution of individual staff or services in supporting personal recovery (INSPIRE and RECOLLECT); five measures met the extended eligibility criteria (RKI; RAQ, ARQ, PERS, and RPRS), and three measures that had been included in the Williams and colleagues review have since been translated, validated and psychometrically tested for use in different settings and populations (REE, RSA and ROSA).

Two measures were rated by service users only (INSPIRE, REE), four measures were only rated by service providers (PERS, RKI, RAQ, RPRS), and four measures had versions for different types of stakeholders

(ROSA: service provider and service user versions); ARQ (service user, carer, service provider versions); RSA

(Person-in-recovery, significant other, service provider, service director versions).

The 26 included articles consisted of seven articles on the RSA, which is the most widely used measure [45, 41–43, 40, 44, 39]. The RSA has versions for four types of stakeholders, and since 2012 it has also been most frequently translated or adapted. For example, there is now a Brief Version of the RSA (RSA-B), Revised RSA (RSA-R), and a version for registered nurses (RSA-RN). It has been used in different countries such as Hong Kong (RSA – HK), a Chinese RSA Service User version (CRSA-SU) and a Persons in Recovery version Malay RSA (PIRV- RSA). It has also been psychometrically tested in Canada and named as the Revised RSA (RSA-R) [43] and in Sweden, (RSA-S) [44]. Of the remaining 18 articles, five were on the RPRS [49–53], three on INSPIRE [31, 54, 55]); there were two articles each on RKI [56], [57], RAQ [58, 59], and REE [60, 61]. Finally, there was one paper on PERS [62], ARQ [63] RECOLLECT [32] and ROSA [38].

These measures were evaluated in relation to the extent to which they assessed support for personal recovery using a conceptual framework of recovery, how they conceptualised recovery-orientation of mental health services and staff, and published data on their psychometric properties.

### Conceptualising recovery

As with the Williams and colleagues review, coverage of the recovery processes of connectedness, hope, identity, meaning and purpose, and empowerment (CHIME) was evaluated for each measure. The aim was to investigate how conceptualisations of recovery, used in identified measures, fit with a robust conceptual framework of recovery, and to be consistent with the approach used in the original review. It is recognised that not all aspects of personal recovery would necessarily need to be supported by mental health services and staff, as these decisions are best shared with and tailored to the needs and wishes of mental health service users of that service.

The measure which most closely matched the CHIME framework in terms of the five recovery processes being comprehensively covered was the INSPIRE measure, which was specifically designed to have an even distribution of items for each recovery process as the CHIME framework was used as the theoretically underpinning during development of this measure of recovery support. Six measures had at least one item per recovery process, but with an uneven distribution of items. For example, most RSA items were on Empowerment, followed by Connectedness and Meaning in life, the REE measure had a higher proportion of items on Connectedness and Empowerment, the ARQ measure had a majority of items related to Connectedness, Identity and Empowerment. The ROSA measure had nearly half of items on Meaning in life and majority of the RECOLLECT fidelity items related to Empowerment. The RKI covered all processes. In the RKI, the items that were not allocated to the recovery processes, some fitted another over-arching theme within the conceptual framework of personal recovery, namely characteristics of recovery journey, for instance, viewing recovery as a ‘struggle’, an ‘individual and unique process’, or a ‘non-linear process’. Some items mapped onto concepts such as ‘functional’ recovery or ‘clinical’ recovery. Three measures did not cover all the recovery processes. The RPRS measure had an equal distribution across the recovery processes of Hope, Identity, Meaning in life and Empowerment, but there were no specific items on Connectedness. The RAQ and PERS items only matched with two recovery processes (Hope and Empowerment) and (Connectedness and Meaning in life) respectively.

### Conceptualising recovery-orientation of services and staff

The conceptualisation of recovery-orientation of services and staff was explored against the inventory of transformation characteristics for a recovery- oriented system of care to see how the measures had defined and operationalised this concept, within four over-arching characteristics of recovery-oriented services. Firstly, the extent to which mental health services are recovery-oriented, is reflected in the attitudes and behaviour of healthcare staff towards service users. This was operationalised as staff knowing what (personal) recovery is and demonstrating through their attitudes and actions that they viewed service users as unique individuals who they believed would recover. This was captured most successfully by three or the four measures (RECOLLECT, RSA and ROSA). Secondly, the need to involve service users in the design, delivery and evaluation of care, was operationalised and measured most comprehensively by the RSA, with multiple items addressing some aspects such as service users having their own opinion and the views of their families sought, as well as having a voice in the system. Thirdly, operationalising the way in which care is planned, delivered, and continually improved was most complex and multi-faceted, involving the ways in which staff and services instil hope and support the development of a meaningful and satisfying life within the community. This also involved services being organised around what service users feel is important to them and attending to aspects like their basic needs, physical health, social support, social relationships. The RSA and RECOLLECT items particularly focussed on ways to support service users build a life within their community. Finally, the ways in which transformation is led and managed was only partially captured in some items, usually in an implicit rather than explicit way, for example with items measuring the extent to which services enabled shared decision making or recognised the importance of responding to cultural diversity.

The aspects of recovery-oriented services which were not captured within these measures were activities focussed around enabling citizenship such as collaborating with service users in addressing social and economic barriers and determinants of health. In a recent critical conceptual review, the ‘silence’ of the personal mental health recovery literature on the impact of various socio-structural inequalities on the recovery process has been noted [14].

### Psychometric properties

The INSPIRE had the most extensive psychometric testing, having used a range of reliability, validity and sensitivity to change assessments. At least one version of INSPIRE (Brief INSPIRE, INSPIRE-O or Full INSPIRE) has been translated into 23 languages, with the full version of INSPIRE being available in 13 languages (see *)^2^. The RSA is most widely used and tested for use in different populations, particularly outside the country of origin.

### Strengths and limitations

A key strength of this present review is that it provides a follow up of the previous systematic review of personal recovery orientation of mental health services, conducted over a decade ago. The study was conducted according to the Preferred Reporting Items for Systematic Reviews and Meta-Analyses (PRISMA) [64] methodology for conducting a systematic review, which has the advantages of providing clarity and transparency of reporting, permits replicability and allowing the strengths and weaknesses of the review to be more readily assessed. The systematic search used Covidence software, which had the benefits of being able to conduct key stages of the review more rapidly and to collaborate with colleagues more easily when screening for eligibility criteria, for example conducting double ratings of eligibility for inclusion at both title/abstract and at full text screening stages and identifying papers where the eligibility was unclear and further discussion was necessary. To the best of our knowledge, this review serves as the first review of literature on measures of the recovery orientation of mental health services and staff which examines the way measures have conceptualised both recovery and recovery-oriented practice.

There are several limitations. First, it should be noted that potentially useful measures were excluded based on non-availability. Second, staff rated measures of recovery-orientation of mental health services, recovery knowledge, attitudes, recovery-promoting relationships and competencies that were created before 2012 but where there have been no publications reporting further developments, adaptations, or psychometric testing since will have been missed. Third, the exclusion of non-English studies may have missed important measures in other languages. Fourth, the CSA, Illumina and TRIP databases searched in this review were not included,

Fifth, there was no available validated quality appraisal tool, with a scoring scheme to assess the quality of the measures included in the present review. Therefore, we could not assess the reporting quality of the identified studies. Lastly, the use of the inventory of transformation characteristics for recovery-oriented system of care led to some difficulties in matching items. Those items on measures with same or very similar wording to the inventory items were easier to fit, whereas for some items the fit was implicit rather than explicit. The judgement of how individual items in the measures mapped onto the recovery and recovery-oriented practice frameworks was conducted by two raters, rather than four raters use for rating in the Williams et al review for rating items mapping onto the CHIME recovery framework, so could have increased allocation errors.

#### Research implications

The key research implication emerging from this review is that there remains a lack of a single gold-standard measure of recovery-orientation of services. This would be a measure that satisfies the criteria for being psychometrically valid and reliable, sensitive to change, and easy to use, and has a good fit with both conceptualisations of personal recovery and recovery-oriented services and/or systems. This knowledge gap could be filled through the development of a new measure which uses an underpinning conceptual model or framework of recovery-oriented services and/or systems to ensure items have a good fit and there is a comprehensive coverage of items across all domains of interest. Potentially, such a measure could include different versions to be completed by relevant stakeholders (for instance, senior leaders/ directors, frontline team/ service managers, clinical staff, service users and carers) which reflect different levels of an organisation/or service or system. It could also use more than one framework of recovery-oriented services and/or systems to generate an initial pool of items, to ensure full coverage of all relevant domains, for example Boutillier and colleagues [19] and Australian mental health framework [24].

### Clinical implications

New evidence presented in this review suggests that there are several recovery measures available that show some promise for use in routine clinical practice assessment use. For example, the original 28 item INSPIRE measure (28 items) now has a Brief INSPIRE version (with 5 items), similarly, the RSA (36 items), has a Brief RSA (RSA-B, 12 items). The RSA was adapted and renamed Recovery-Oriented Services Assessment (ROSA), lowering the readability age and length of the measure to 15 items, making them all easier to use.

## Conclusions

This review has updated and extended the Williams and colleagues review, which identified three main knowledge gaps, firstly that there was not a single gold-standard measure of recovery-orientation of services, secondly, that there was no single measure which showed a good fit with the conceptual framework of recovery, and thirdly, that none of the measures showed adequate reliability or sensitivity to change.

After over a decade of research in the field of recovery outcome measurement, the first knowledge gap has yet to be filled. Whilst there is still a long way to go, some steps have been taken to develop new, or adapt existing recovery measures so they have been either developed and/or tested in different populations/ countries and are more culturally sensitive. There is a need to adapt measures for use with minority populations within certain countries, for example black African service users in the UK [65, 66].

With respect to the second knowledge gap, the measure which showed the best fit with CHIME framework in terms of the five recovery processes being comprehensively covered was the INSPIRE measure, which was specifically designed to have an even distribution of items for each recovery process as the CHIME framework was used as the theoretically underpinning during development of this measure of recovery support.

Finally, in relation to the third knowledge gap, often sensitivity to change for measures was not reported. The INSPIRE was the only measure that showed both an adequate sensitivity to change and reliability.

### Electronic Supplementary Material

Below is the link to the electronic supplementary material

Supplementary Material 1

## Data Availability

Not applicable

## Footnotes

1 Personal email correspondence with the author, Prof Mary Chambers on 24.8.22

2 Bosnian, Catalan*, Czech*, Danish*, Dutch*, Estonian*, Finnish*, French, German, Gujarati, Hebrew, Italian*, Japanese*, Luganda, Norwegian*, Russian*, Slovene, Spanish*, Swahili, Swedish*, Thai, Traditional Chinese*. Arabic and Portuguese (coming soon).
