# Supplementary material for: A systematic review of measures of the personal recovery orientation of mental health services and staff
Source: Int J Ment Health Syst. 2023 Oct 17;17:33. doi: 10.1186/s13033-023-00600-y (PMC10580616; doi:10.1186/s13033-023-00600-y)

**Supplementary file: Leamy et al. Systematic review of recovery-oriented measures**

Recovery OR personal recovery OR wellness OR mental well-being OR recovery orientation OR recovery promotion

AND

Mental illness OR mental disorder* OR psychiatric disease OR psychiatric disorder* OR psychiatric illness OR chronic mental illness OR affective disorder* OR mood disorder* or psychosis

AND

Instrument outcome assessment OR outcomes research OR measurement OR process assessment OR outcome assessment OR treatment effectiveness evaluation OR treatment outcomes OR questionnaire* OR rating OR scale* or standardised test* OR survey* or instrument* or inventor* OR index OR assessment* OR profile OR reliability OR validity OR soundness OR evaluat$ or assess$ or test$ or pilot OR develop$ OR construct$ OR correlation OR gold standard OR psychometric adj characteristics OR internal adj consistency OR alpha OR beta OR cronbach OR create$ OR scale adj develop$ OR stability OR test adj construction OR validation adj construction OR validation OR shorten OR modify OR compar$ OR adapt OR revis$ OR alter OR increase OR improve OR design

Limit to human and English language and tear 2012- current (which was march 2021)


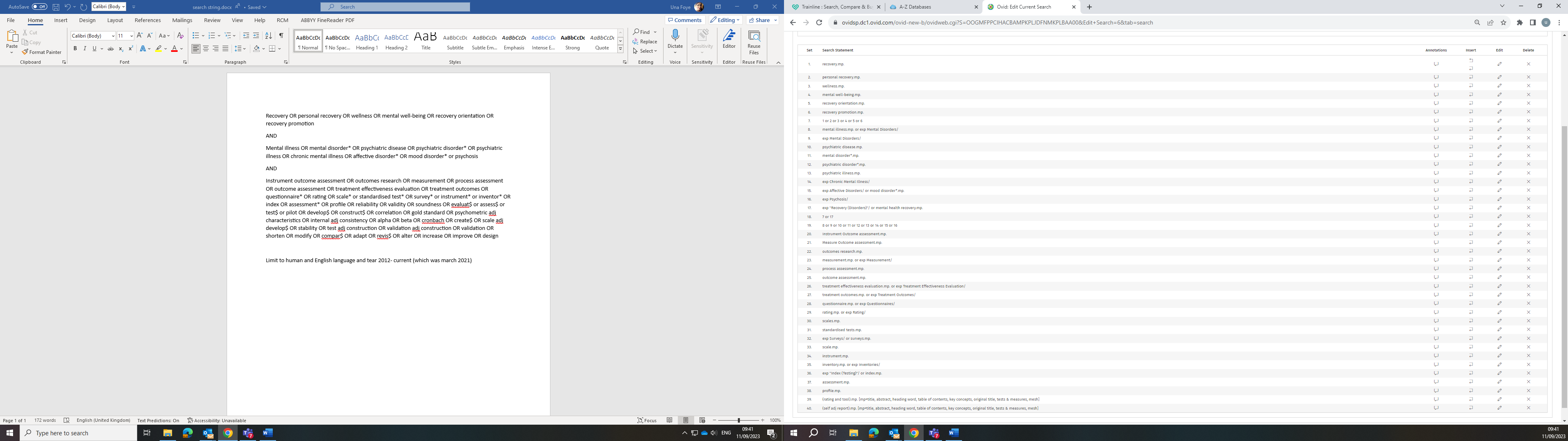

Supplement: Supplementary file 1 — Supplementary Material 1 [file 13033_2023_600_MOESM1_ESM.docx]
